# Racism in obstetric care: a psychometric study of the Gendered Racial Microaggressions Scale among Global Majority birthing people in obstetric contexts 

**DOI:** 10.1186/s12884-024-06642-5

**Published:** 2024-06-28

**Authors:** Frances M. Howell, Katharine J. McCarthy, Natalie Boychuk, Micki Burdick, Sarah Nowlin, Sheela Maru, Oluwadamilola Oshewa, Maria Monterroso, Alva Rodriguez, Cecilia Katzenstein, Regina Longley, Camila Cabrera, Elizabeth A. Howell, Lisa Levine, Teresa Janevic, Daniel A. Gundersen

**Affiliations:** 1 Department of Population Health Science Policy, Icahn School of Medicine at Mount Sinai, 722 W 168th Street, Room 722, New York, NY 10032 USA; 2 Department of Epidemiology, Columbia University Mailman School of Public Health, 1770 Madison Avenue, 2nd Floor, New York, NY 10029 USA; 3 Department of Obstetrics, Gynecology, and Reproductive Science, Icahn School of Medicine at Mount Sinai, 1770 Madison Avenue, 2nd Floor, New York, NY 10029 USA; 4 Department of Women & Gender Studies, University of Delaware, 25N College Ave. 205 McDowell Hall, Newark, DE 19716 USA; 5 Center for Nursing Research and Innovation, Mount Sinai Health System, 19 East 98th Street, 3rd Floor, Suite E, New York, NY 10029 USA; 6 Department of Health System Design and Global Health, Icahn School of Medicine at Mount Sinai, New York, USA; 7 Arnhold Institute for Global Health, Icahn School of Medicine at Mount Sinai, 1216 Fifth Ave, 5th Floor, New York, NY 10029 USA; 8 New York City Health + Hospitals/Elmhurst, 1216 Fifth Ave, 5th Floor, New York, NY 10029 USA; 9 Department of Obstetrics and Gynecology, University of Pennsylvania, 3400 Spruce Street, Philadelphia, PA 19104 USA; 10 Icahn School of Medicine at Mount Sinai, Box 1199, One Gustave L. Levy Place, New York, NY 10029 USA; 11 Department of Obstetrics, Gynecology, and Reproductive Science, Icahn School of Medicine at Mount Sinai, Box 1199, One Gustave L. Levy Place, New York, NY 10029 USA; 12 Department of Obstetrics and Gynecology, University of Pennsylvania, 3400 Spruce Street, 5 Dulles, Philadelphia, PA 19194 USA; 13 Department of Obstetrics and Gynecology, University of Pennsylvania, 3400 Spruce Street, 2 Silverstein, Philadelphia, PA 19104 USA; 14 Department of Social and Behavioral Sciences, Harvard TH Chan School of Public Health, Boston, USA; 15 Rutgers Institute for Nicotine and Tobacco Studies, Rutgers the State University of New Jersey, 303 George Street, Suite 500 New, Brunswick, NJ 08901 USA; 16 Present Address: New York, USA; 17 Philadelphia, USA

**Keywords:** Gendered racial microaggressions, Measure and assessment development, Intersectionality, Postpartum health, Mental health, Health equity

## Abstract

In the United States, maternal health inequities disproportionately affect Global Majority (e.g., Asian, Black, and Hispanic) populations. Despite a substantial body of research underscoring the influence of racism on these inequities, little research has examined how experiences of gendered racial microaggressions during pregnancy and birth impact racially and ethnically diverse Global Majority pregnant and birthing people in obstetric hospital settings. We evaluated the psychometric properties of an adapted version of Lewis & Neville’s Gendered Racial Microaggressions Scale, using data collected from 417 Global Majority birthing people. Findings from our study indicate that our adapted GRMS is a valid tool for assessing the experiences of gendered racial microaggressions in hospital-based obstetric care settings among Global Majority pregnant and birthing people whose preferred languages are English or Spanish. Item Response Theory (IRT) analysis demonstrated high construct validity of the adapted GRMS scale (Root Mean Square Error of Approximation = 0.1089 (95% CI 0.0921, 0.1263), Comparative Fit Index = 0.977, Standardized Root Mean Square Residual = 0.075, log-likelihood c2 = -85.6, df = 8). IRT analyses demonstrated that the unidimensional model was preferred to the bi-dimensional model as it was more interpretable, had lower AIC and BIC, and all items had large discrimination parameters onto a single factor (all discrimination parameters > 3.0). Given that we found similar response profiles among Black and Hispanic respondents, our Differential Item Functioning analyses support validity among Black, Hispanic, and Spanish-speaking birthing people. Inter-item correlations demonstrated adequate scale reliability, α = 0.97, and empirical reliability = 0.67. Pearsons correlations was used to assess the criterion validity of our adapted scale. Our scale’s total score was significantly and positively related to postpartum depression and anxiety. Researchers and practitioners should seek to address instances of gendered racial microaggressions in obstetric settings, as they are manifestations of systemic and interpersonal racism, and impact postpartum health.

## Introduction

The United States (U.S.) is facing a maternal mortality and morbidity crisis marked by inequities stratified along lines of race, class, gender, and nativity [1]. Research suggests that the rate of severe maternal morbidity has increased over the last few decades, and Asian, Black, and Hispanic pregnant and birthing people are at higher risk compared to white birthing people [2–4]. In New York City (NYC), rates of severe maternal morbidity have increased among Black, and Hispanic pregnant and birthing people, and Southeast Asian pregnant and birthing people are also at high risk [2, 3, 5]. Moreover, Black, Hispanic, and Asian pregnant and birthing people are more likely to have preexisting conditions, such as hypertension and diabetes, which can lead to complications during pregnancy and birth, and influence the likelihood of maternal morbidity and mortality [6]. Thus, pregnant and birthing people of the Global Majority are adversely affected by maternal health inequities in the U. S. [5].

Maternal and reproductive health equity scholars have sounded the alarm that racism, not race, is the underlying cause of these inequities experienced by the Global Majority birthing people. Since then, there has been an increase in scholarship on multiple levels of racism on adverse obstetric outcomes among Black birthing people [7–12]. Nonetheless, research tends to be isolated by a single race or ethnicity and often focuses on comparing adverse maternal health outcomes of Global Majority pregnant and birthing people to white pregnant and birthing people. Taken together, racially, ethnically, and socioeconomically diverse Global Majority pregnant and birthing people experience gendered and racialized discrimination, at multiple levels (structural/systemic, interpersonal, and internal), within healthcare systems. Therefore, our current study takes an intersectional approach to understanding experiences of gendered racial microaggressions in medicalized obstetric contexts.

Intersectionality is a theoretical and analytic framework that acknowledges that no person has an essential single social identity, but rather multiple identities are experienced simultaneously and reflect interlocking systems of privilege and oppression [13–15]. Using intersectionality as a central framework for this study, we examine whether racially and ethnically diverse groups of Global Majority birthing people experience gendered racial microaggressions during pregnancy and birth in hospital and medical-based obstetric settings. Microaggressions are subtle forms of racism that include subtle verbal and physical insults, as well as ignoring) which in turn may contribute to maternal health inequities [16, 17]. Building on this definition of microaggressions, Lewis et al., used intersectionality to conceptualize and define gendered racial microaggressions as “subtle and everyday verbal, behavioral, and environmental expressions of oppression based on the intersection of one’s race and gender [18]”. Even though Lewis et al. focused on Black women’s unique experiences with gendered racial microaggressions and its effect on mental health, the present study applies this concept to all Global Majority pregnant and birthing people [18].

Pregnant and birthing people of the Global Majority, from varying socioeconomic statuses, are affected by racist and sexist prejudices and discrimination in obstetric settings. For example, researchers found that stereotypes based on gendered racism (such as being a single-parent and sexually promiscuous) are associated with stress during pregnancy, a known contributor to adverse birth outcomes among Black and Hispanic birthing people of low socioeconomic status [19]. Additionally, a qualitative study found that Asian, Black, Hispanic, and Middle Eastern birthing people of various socioeconomic statuses reported experiencing racism in their healthcare interactions, which negatively impacted their mental health during pregnancy [20]. In the context of pregnancy and birth, gendered racial microaggressions, specifically, can materialize as having comments on birth plans and care concerns be ignored, or feeling disrespected and excluded from pregnancy and postpartum-specific resources, for example. Furthermore, experiencing gendered racial microaggression may also negatively impact the mental health of pregnant and birthing Global Majority individuals. Research demonstrates that among Global Majority people, experiences of gendered racial microaggressions are associated with symptoms of anxiety and depression [21, 22]; disordered eating, body shaming, and emotional dysregulation [23]; and are related to discrimination based on racism, sexism, level of education, and nativity [24]. Despite the evidence demonstrating the overall mental health impact of gendered racial microaggressions on Global Majority individuals, to our knowledge, little is known about how this literature extends to gendered racial microaggressions in the uniquely situated context of obstetric care. However, as demonstrated by Crawford et al., there is a lack of consensus on exactly how perinatal and maternal health equity researchers can best capture instances of microaggressions in obstetric care [25]. To our knowledge, there is also a dearth of research on the intersectional impact of gendered racial microaggressions, on adverse perinatal and postpartum outcomes among a diverse Global Majority pregnant and birthing people. Given that hospitals and healthcare systems serve a diverse birthing population and not a singular race or ethnicity, a measure that captures racially diverse experiences of gendered racial microaggressions through an intersectional lens in obstetric settings within the Global Majority that can be administered to all patients is greatly needed. Therefore, in the current study, we have adapted the Gendered Racial Microaggressions Scale (GRMS) for the context of obstetric care during pregnancy and birth in hospital and medical settings and evaluated its psychometric properties among English- or Spanish-speaking Global Majority birthing people [18].

### Current study

The GRMS [18] is commonly used to measure the frequency of gendered racial microaggressions experienced by Black women, specifically. However, to our knowledge, there has yet to be a measure that captures the frequency of gendered racial microaggressions experienced among a diverse sample of Global Majority people, nor in the context of pregnancy, birth, and postpartum. We are unaware of studies that have used the GRMS in any language other than English. Therefore, we sought to evaluate the psychometric properties of a revised version of Lewis & Neville’s GRMS adapted to a Global Majority population within obstetrics settings [18]. In the present study, we examined whether our adapted measure accurately measures the frequency of gendered racial microaggressions among Global Majority birthing people during pregnancy and birth in both English and Spanish. This study evaluates the psychometric properties of our adapted measure by conducting item response theory (IRT) to assess the construct validity, differential item functioning to evaluate the performance of individual items, and computed Pearson correlations, between our adapted GRMS scale with postpartum depression and anxiety scales, to assess the criterion validity.

## Methods

### Procedure

**Fig. 1:**
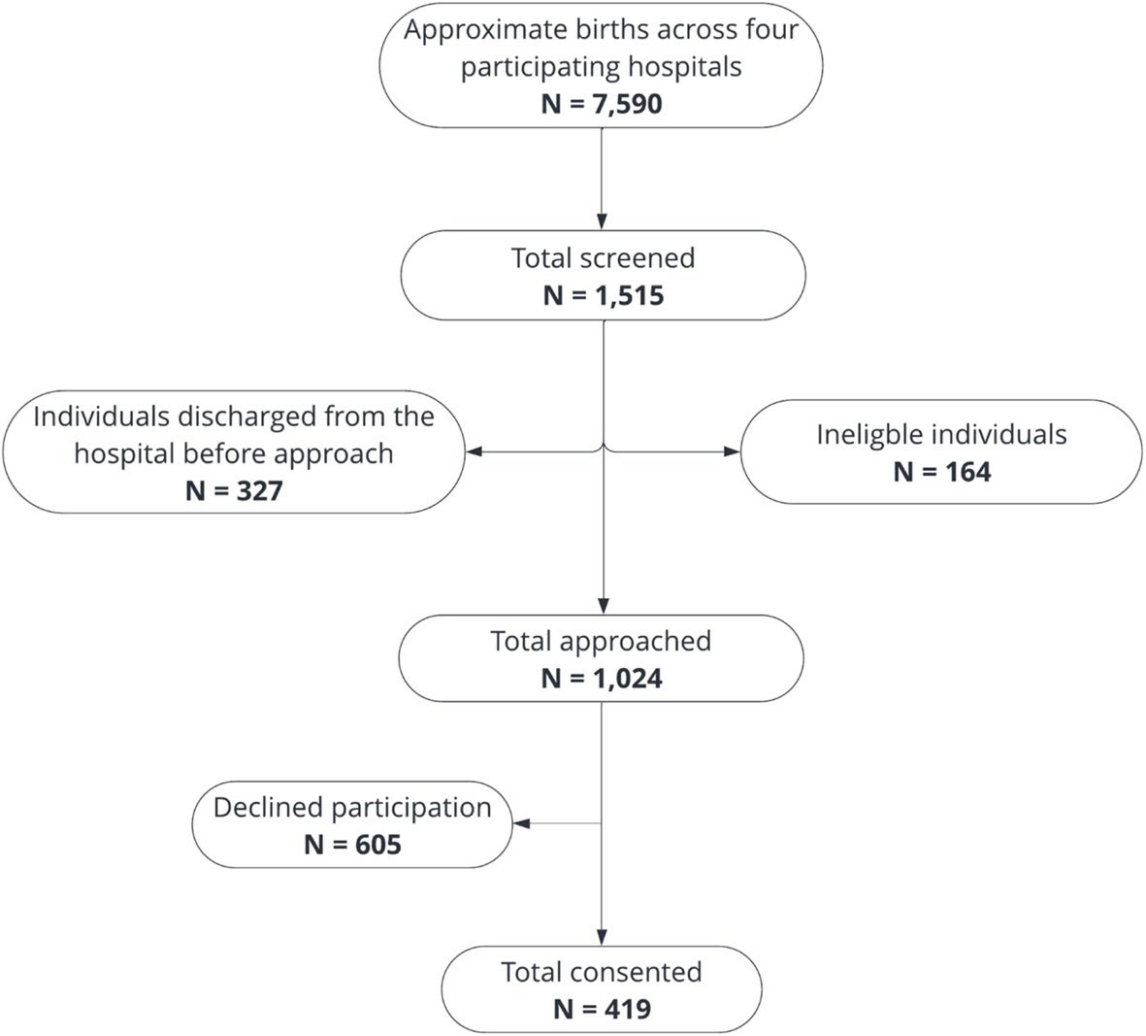
Flow chart of participant recruitment

This validation study was part of a larger multi-site study that examined the association between experiencing multiple levels of racism [12], childbirth experiences, postpartum health outcomes, and COVID-19-related economic stress among patients who delivered in a hospital setting. Participants were recruited, in the postpartum unit before discharge, from four hospitals in New York City and Philadelphia between March 2022 and March 2023. Individuals who identified as Asian, Black, or Hispanic, recently gave birth, spoke either English or Spanish and had access to a smart cell phone were considered eligible for participation. Potential participants were approached at their bedside, in the postpartum unit, to gauge their interest in the study, and were asked to confirm whether the race and ethnicity reported in their hospital records aligned with their self-identified race, ethnicity, and preferred language before enrolling in the study. A final sample of 420 postpartum Global Majority birthing people. Please refer to Fig. 1 for a flow chart of participant recruitment. Every eligible participant in this research study provided their informed consent before being enrolled in the study. After obtaining informed consent, participants were asked to complete an electronic survey in either English or Spanish before they were discharged from the hospital. The electronic survey was hosted on Research Electronic Data Capture (REDCap), a secure and HIPAA-compliant data collection platform, hosted at one of the participating institutions [26, 27]. The Spanish version of the survey was translated from English by a professional translation service and two Spanish-speaking members of the research team reviewed the translation to ensure accuracy. Participants were compensated up to a total of $100 for completing all research activities within the 90-day study period. A large non-profit teaching hospital in the Northeast granted institutional review board approval.

### Measures

#### Gendered racial microaggressions scale

In this study, we revised and adapted the GRMS: a validated, 26-item, to assess the frequency (0 = *never to* 5 = *once a week or more*) of gendered racial microaggressions experienced by our participants [18]. The GRMS is broken into four factors that capture the experiences of gendered racial microaggressions that are specific to Black women related to negative and harmful racialized and cisgender stereotypes [18]). The four factors are Factor A: Assumptions of Beauty and Sexual Objectification; Factor B: Silenced and Marginalized; Factor C: Strong Black Woman Stereotype; and Factor D: Angry Black Woman Stereotype. Overall, each factor assesses the frequency and stress of experiencing gendered racial microaggressions, in personal and professional contexts, that are associated with negative stereotypes associated with Black womanhood. The GRMS has previously demonstrated good validity and reliability for frequency (α = 0.92) among Black women [18].

The study team created the adapted GRMS in collaboration with our community advisory board (VIBE Community Working Group; CWG). The CWG was comprised of eight racially and ethnically diverse members (recruited by a research member in New York City and Philadelphia, respectively) consisting of doulas, community health workers, educators, OB/GYNs, and reproductive justice advocates. There was an equal collaboration between us researchers and the CWG on revising the GRMS frequency scale to measure experiences of gendered racial microaggressions within obstetric settings among a Global Majority population whose preferred language was either English or Spanish. During our CWG meetings, we had in-depth discussions on the scale and reached a consensus on which items should be included, or excluded, and how to adapt the phrasing of specific items. First, the CWG recommended that we update the prompt at the beginning of the survey to have participants think about experiencing gendered racial microaggressions within the context of pregnancy and hospital birth. In this way, instead of opening our adapted GRMS frequency scale with the original GRMS prompt of, “Using both a stress appraisal scale (Range: 0 (*not at all stressful*) to 5 (*extremely stressful*)) and a frequency scale (Range: 0—(*never*) to 5 (*once a week or more*)) rate how often you experience each event in your lifetime” our version began with “Using a frequency scale, range 0 (never) to 5 (all the time), thinking about over the course of your pregnancy care and delivery care in the hospital, rate the following”. See Table 1 for a comparison of the original items on the GRMS and our adapted version.

**Table 1:** Comparison of the GRMS and the adapted GRMS scales and scale frequency

Original GRMS Survey Item	Adapted GRMS Survey Item
**Factor A: Assumptions of Beauty and Sexual Objectification**	
Unattractive because of size of butt^a^	
Negative comments about size of facial features^a^	
Imitated the way they think Black women speak^a^	
Someone made me feel unattractive^a^	
Objectified me based on physical features^a^	
Someone assumed I have a certain body type^a^	
Made a sexually inappropriate comment^a^	
Negative comments about my hair when natural^a^	
Assumed I was sexually promiscuous^a^	
**Factor B: Silenced and Marginalized**	
I have felt unheard	I have felt unheard
My comments have been ignored	My comments have been ignored
Someone challenged my authority	Someone challenged my autonomy^b^
I have been disrespected in the workplace	I have been disrespected^b^
Someone has tried to “put me in my place”	Someone has tried to “put me in my place”
Felt excluded from networking opportunities	Felt excluded from services or resources^b^
Assumed I did not have much to contribute to the conversation	Assumed I did not have much to contribute to the conversation regarding my care^b^
**Factor C: Strong Black Woman Stereotype**	
Someone assumed I was sassy and straightforward have^a^	
I have been told that I am too independent^a^	
Someone made me feel exotic as a Black woman^a^	
I have been told that I am too assertive^a^	
Assumed to be a strong Black woman^a^	
**Factor D: Angry Black Woman Stereotype**	
Someone told me to calm down	Someone told me to calm down
Perceived to be “angry Black woman”^a^	
Someone accused me of being angry when speaking calm	Someone accused me of being angry when speaking assertively^a^

^a^Reflects survey items that were not included in our adapted version of the scale

^b^Reflects surveys items that have been adapted to obstetric context

Second, the research team consulted the CWG’s expertise on working with birthing people of varying social identities (e.g., races, ethnicities, socioeconomic statuses, and nativity) on how which factors to include in our adapted scale and which items best capture experiences of gendered racial microaggressions among not just Black women, but Global Majority pregnant and birthing people of various races and ethnicities. The CWG and research team reviewed the four factors in the original scale and agreed that Factor B: Silence and Marginalization best represented the wide range of Global Majority pregnant birthing people’s experiences of obstetric racism. We also agreed that Global Majority pregnant and birthing people experience similar cultural stereotypes within obstetric settings at the intersection of gender, race, and ethnicity. We reached a consensus on including three items from Factor D: Angry Black Woman Stereotypes to our study’s adapted GRMS scale. For example, we included “Someone told me to calm down”, and changed “Someone accused me of being angry when speaking” ‘calmly’ to ‘assertively’. Our decision to include items from Factor D: Angry Black Women Stereotypes in our adapted GRMS is consistent with intersectional research on gender and ethnic stereotypes suggesting that Asian, Black, and Hispanic birthing people are often stereotyped as being loud, unintelligent, hypersexual, and exotic, lower class, and young single mothers [27–29]. We did not include Factor B nor Factor C, and only specific items from Factor D because we thought they were either too specific to Black women’s experiences or were not appropriate for obstetric contexts. Although we recognize that each race and ethnicity in the Global Majority has its own unique gendered and racialized stereotypes, there are commonalities across all Global Majority pregnant birthing people and chose the items that best applied to a diverse multiracial and multiethnic population. Thus, based on both prior theories found in the literature, and the lived experiences of our CWG members, we edited the GRMS according to common gendered racial microaggressions experienced by a diverse population of Global Majority pregnant birthing people in obstetric and hospital-based contexts.

The CWG and research team changed the wording of specific items to better capture experiencing gendered racial microaggressions in obstetric care. For instance, we changed “Assumed I did not have much to contribute to the conversation” to “Assumed I did not have much to contribute to the conversation regarding my care”. The wording changes are supported by previous research on obstetric racism and gendered racism in reproductive healthcare settings. For example, research on Black birthing people’s experiences of obstetric racism and gendered racism has indicated that Black birthing patients reported that their autonomy was violated and undermined during hospital births [30, 31]. Similarly, both Asian and Black birthing people have reported that they feel invisible and silenced because their views and opinions are often ignored, which may lead to their reproductive health concerns being dismissed by their providers [28, 31]. Finally, the CWG and research team met to review our final version of the adapted GRMS to confirm that it reflected both the literature on gendered racial microaggressions among Asian, Black, and Hispanic birthing people and the experiences that the CWG members witnessed as reproductive justice advocates and birth equity workers.

#### Postpartum anxiety

The Generalized Anxiety Disorder (GAD-7) questionnaire was used to assess participants’ symptoms of anxiety [32, 33]. The GAD-7 is a seven-item scale that measures participants’ self-reported anxiety symptoms over the past 2 weeks. Participants indicated the frequency with that they experienced anxiety symptoms using a Likert-type scale from 0 (*not at all*) to 3 *(nearly every day).* Total average scores indicated no (scores of 4 or less), mild (scores of 5 to 9), moderate (scores of 10 to 14), and severe (scores of 15 or greater) levels of anxiety. We used the recommended threshold of 10 or higher to refer participants for additional anxiety evaluation. Prior research has demonstrated the GAD-7 as a useful scale for meaningfully measuring anxiety among pregnant and postpartum individuals in clinical settings and among Global Majority birthing populations whose preferred language is English or Spanish [34–37].

#### Postpartum depression

We assessed postpartum depression using the Edinburgh Postnatal Depression Scale (EPDS) [38]. The EPDS is a 10-item scale designed to assess self-reported postnatal depression symptomatology. Items were rated on a Likert-type scale from 0 (*as much as I always could*) to 3 (*hardly at all*). Participants who scored a 13 or higher (the recommended threshold used to identify depressive symptoms) and/or indicated self-harm and suicidal ideation were referred for additional postpartum depression evaluation and given resources, such as the phone number of the National Suicide Prevention Lifeline helpline and local mental health crisis helplines. Validity of the EPDS has been assessed in over 37 studies, and globally among Asian, Black, and Hispanic Global Majority birthing people [39–41].

### Data analysis

We used item response theory (IRT) to assess the construct validity of the adapted 9-item GRMS, the performance of individual items, and differential item functioning in our study of a population of Black and Hispanic birthing people. Due to the multifactorial structure of the initial scale and reduction in the adapted model [18], we examined dimensionality by comparing the first to second eigenvalues of the correlation matrix, assessed the relative fit and interpretability of unidimensional, bidimensional, and bifactor-graded response models. Model fit was assessed by examining model fit indices (Akaike information criterion [AIC], Bayesian information criterion [BIC], comparative fit index [CFI], root mean square error of approximation] [RMSEA], and standardized root mean square residual [SRMR]), and residual correlations. We compared relative model fit using the log-likelihood ratio test. We also considered model conceptual utility and inspected discrimination parameters and item-and-test-information curves to identify items that distinguished well between perceived discrimination levels and to identify where the scale and items are most informative. The empirical (marginal) reliability and Cronbach’s alpha (internal consistency) were used to assess internal consistency reliability (α > 0.70 considered acceptable). Less than five percent (4.5%) of respondents had missing data on scale items. We performed IRT analysis using the stochastic (MHRM) estimator which accounts for missing data using the full information maximum likelihood method [42].

As an additional step, we tested differential item functioning (DIF) between Black and Hispanic participants, who together comprised over 80% of the study sample, and by preferred survey language (English or Spanish). While participants of all races and or ethnicities were included in the IRT analysis, we were only able to conduct DIF analysis among Black and Hispanic respondents, due to insufficient sample size among Asians and those who did not report their race and ethnicity or listed it as unknown/’other’ [42]. The means and correlations for the adapted GRMS, GAD-7, and EPDS are, however, reported by Asian, Black, and Hispanic participants in Tables 2, 3 and 4. The presence of DIF was assessed using the likelihood ratio test, using the Bonferroni correction to adjust for multiple comparisons. Anchor item selection was informed by scale item frequencies considered similar across subgroups. Given empirical evidence of the impact of gendered racial microaggressions’ on mental health outcomes, as well as the associations between experiences of racism and postpartum depression symptoms, we assessed the criterion validity of the revised GRMS by computing Pearson correlation with the postpartum depression (EPDS) and anxiety (GAD-7) measures [43–45]. Multiple imputation was conducted on the GRMS, GAD-7, and EPDS before correlation analysis to produce 4 complete datasets with 30 imputations and by specifying the conditional distribution for each variable. Results were pooled across completed datasets using Rubin’s rules [46]. Model convergence was assessed by examining the correspondence between estimates across each completed dataset and r(hat) estimates (a convergence statistic). The acceptability of imputed data was assessed visually by inspecting graphics of model residuals, predicted versus expected values, and histograms displaying the frequency of imputed, observed, and total cases to confirm similar distribution of variable frequency and error terms in the original and imputed samples. We conducted psychometric analysis using the “mirt” package, and multiple imputation using the “mi” package in R Studio (Version 2023.03.0 + 38). Data management was performed in SAS (Version 9.4) [46, 47].

**Table 2:** Subgroup analysis, Asian participants - means, standard deviations and correlations among the GRMS, GAD-7, and EPDS measures

Measure	*n*	*M*	*SD*	1	2	3
1. GRMS	43	1.85	3.50	─		
2. GAD-7	43	3.05	2.97	.09	─	
3. EPDS	43	4.82	3.82	.35*	.28	─

*p < 0.05

**Table 3:** Subgroup analysis, Black participants - means, standard deviations and correlations among the GRMS, GAD-7, and EPDS measures

Measure	*n*	*M*	*SD*	1	2	3
1. GRMS	156	2.99	6.90	─		
2. GAD-7	156	3.87	4.41	.33**	─	
3. EPDS	156	4.64	4.12	.20***	.40***	─

***p < .01, ***p < .001

**Table 4:** Subgroup analysis, Hispanic/Hispanic participants - means, standard deviations and correlations among the GRMS, GAD-7, and EPDS measures

Measure	*n*	*M*	*SD*	1	2	3
1. GRMS	159	3.20	6.65	─		
2. GAD-7	159	4.25	4.26	.38***	─	
3. EPDS	159	5.12	4.01	.25***	.55***	─

***p < .001

## Results

### Participants

The sample included 399 participants with a mean age of 30.2 years (*SD*_*age*_ = 6.1). Participants identified as Black (38%) followed by Hispanic (38%), 10.3% as Asian, and 13.6% as ‘Other/ Unknown’. For about half of the participants, the highest level of education was high school/GED, while roughly one-quarter completed at least some college or a technical degree, or were a college graduate or higher, respectively. More than half (53%) reported an income of less than $25,000 per year and a similar proportion (53%) were non-native to the U.S. Most participants completed the survey in English (73.5%) and all participants who completed the survey in Spanish self-identified as Hispanic.

### Descriptive statistics

Of the 417 participants who completed the survey, 18 (4.5%) did not complete scale items and were excluded from the GRMS psychometric analysis (analytic *N* = 399). Nearly 2 in 5 (38.7%) of the sample endorsed at least one experience of gendered racial microaggressions. The most highly endorsed scale item was feeling “unheard” (21% experienced at least once), followed by “my comments have been ignored” (18% experienced at least once). The least commonly endorsed item was feeling “excluded from services or resources” (8%). We observed less variation in the frequency of the above items, with most respondents indicating “never” experiencing the event, followed by “rarely” experiencing the event. We decided not to collapse scale frequency categories before the IRT analysis because they reflect important distinctions in the degree of exposure to and salience of gendered racial microaggressions in obstetric settings.

### Reliability

Inter-item correlations for the entire sample and racial and ethnic participant subgroups are shown in Table 5. For the full scale, α = 0.97 and empirical reliability = 0.67, demonstrating adequate scale reliability.

**Table 5:** Adapted GRMS frequency scale items, correlations, means and standard deviations

Item	1	2	3	4	5	6	7	8	9	*M*	*SD*
*Full Sample*											
1. I have felt unheard	1.0									0.59	1.20
2. My comments have been ignored	0.92	1.0								0.49	1.11
3. Someone challenged my autonomy	0.75	0.82	1.0							0.29	0.89
4. I have been disrespected	0.76	0.78	0.80	1.0						0.31	0.93
5. Someone has tried to “put me in my place”	0.75	0.79	0.87	0.92	1.0					0.24	0.82
6. Felt excluded from services or resources	0.74	0.81	0.80	0.87	0.86	1.0				0.26	0.87
7. Assumed I did not have much to contribute to the conversation regarding my care	0.78	0.80	0.78	0.86	0.88	0.86	1.0			0.30	0.92
8. Someone told me to calm down	0.64	0.70	0.74	0.76	0.83	0.71	0.76	1.0		0.45	1.07
9. Someone accused me of being angry when speaking assertively	0.67	0.72	0.79	0.79	0.86	0.73	0.78	0.83	1.0	0.29	0.93
*Asian Participants*											
1. I have felt unheard	1.0									0.32	0.65
2. My comments have been ignored		1.0								0.32	0.72
3. Someone challenged my autonomy	0.53	0.64	1.0							0.17	0.50
4. I have been disrespected	0.73	0.66	0.26	1.0						0.09	0.37
5. Someone has tried to “put me in my place”	0.63	0.58	0.58	0.79	1.0					0.10	0.38
6. Felt excluded from services or resources	0.63	0.58	0.58	0.95	0.73	1.0				0.10	0.37
7. Assumed I did not have much to contribute to the conversation regarding my care	0.95	0.96	0.75	0.82	0.76	0.76	1.0			0.22	0.62
8. Someone told me to calm down	0.51	0.48	0.35	0.49	0.77	0.59	0.31	1.0		0.34	0.88
9. Someone accused me of being angry when speaking assertively	0.50	0.46	0.50	0.41	0.68	0.69	0.10	0.96	1.0	0.20	0.72
*Black Participants*											
1. I have felt unheard	1.0									0.53	1.10
2. My comments have been ignored	0.93	1.0								0.41	1.00
3. Someone challenged my autonomy	0.86	0.90	1.0							0.22	0.74
4. I have been disrespected	0.78	0.77	0.75	1.0						0.38	1.00
5. Someone has tried to “put me in my place”	0.78	0.81	0.86	0.93	1.0					0.28	0.90
6. Felt excluded from services or resources	0.81	0.88	0.94	0.82	0.88	1.0				0.28	0.86
7. Assumed I did not have much to contribute to the conversation regarding my care	0.78	0.81	0.79	0.89	0.88	0.85	1.0			0.28	0.88
8. Someone told me to calm down	0.64	0.75	0.72	0.81	0.88	0.84	0.88	1.0		0.37	0.93
9. Someone accused me of being angry when speaking assertively	0.74	0.78	0.80	0.88	0.90	0.83	0.87	0.94	1.0	0.25	0.82
*Hispanic Participants*											
1. I have felt unheard	1.0									0.57	1.23
2. My comments have been ignored	0.90	1.0								0.48	1.12
3. Someone challenged my autonomy	0.71	0.78	1.0							0.32	0.90
4. I have been disrespected	0.70	0.75	0.89	1.0						0.24	0.82
5. Someone has tried to “put me in my place”	0.64	0.70	0.91	0.87	1.0					0.18	0.68
6. Felt excluded from services or resources	0.66	0.73	0.69	0.81	0.73	1.0				0.22	0.82
7. Assumed I did not have much to contribute to the conversation regarding my care	0.76	0.75	0.81	0.79	0.83	0.82	1.0			0.30	0.95
8. Someone told me to calm down	0.57	0.63	0.79	0.72	0.79	0.50	0.62	1.0		0.55	1.17
9. Someone accused me of being angry when speaking assertively	0.61	0.68	0.82	0.77	0.79	0.63	0.74	0.87	1.0	0.33	0.98

*N* = 399. Item scale range: *(0* = *never to 5* = *all the time)*; Polychoric correlations reported. Empirical reliability = 0.67

### IRT analysis

**Fig. 2:**
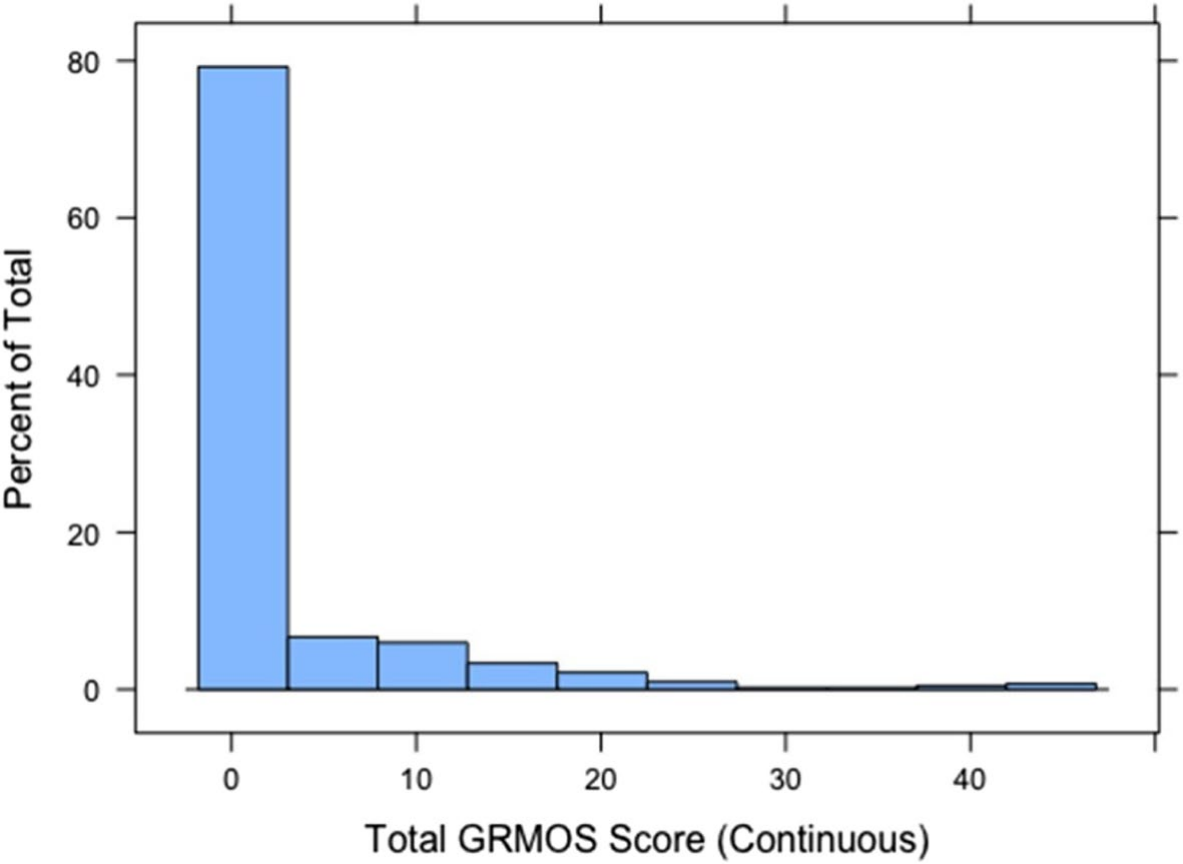
Histogram of total GRMOS scale summed (continuous)

**Fig. 3:**
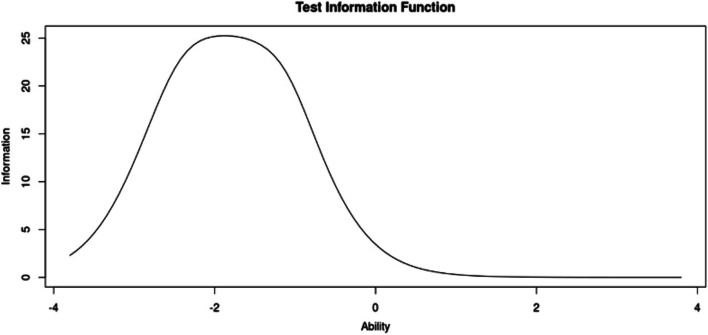
GRMOS test information (**A**) and item information curves (**B**)

We assessed the construct validity of the adapted nine-item GRMS frequency scale using IRT by imposing a unidimensional and bi-dimensional model structure. We hypothesized that the scale would map onto one of these functional forms given that eight items were drawn from one of the original GRMS scale factors, and two items were adapted from a second factor. The first Eigenvalue was 2.8 times greater than the second. IRT analyses demonstrated that the unidimensional model was preferred to the bi-dimensional model as, although it had some residual correlation between scale items (> 0.10), was more interpretable and had lower AIC and BIC. Moreover, while the two-factor structure demonstrated a slightly improved model fit, as shown in Table 6, the degree of improvement was negligible, and the solution was less interpretable than the one-factor solution. In the two-factor model, two items had large discrimination parameters (Items 2 and 5 with discrimination parameters > 7.0) whereas in the one-factor solution, all items had large discrimination parameters onto a single factor (all discrimination parameters > 3.0), as shown in Table 7. The overall model fit of the one-factor nine-item GRMS graded response model was RMSEA = 0.1089 (95% CI 0.0921, 0.1263), CFI = 0.977, SRMS = 0.075, log-likelihood χ^2^ = -85.6, *df* = 8. However, some residual correlation (> 0.10) was apparent in the one-factor model, and we compared discrimination parameters to a general factor in a bi-factor model with a targeted rotation. Comparison to the bifactor model allows examination of whether there are likely to be residual dependencies between subgroups of items within the scale and the impact of those residual dependences on discrimination parameters by comparing the discrimination parameters from a unidimensional model and the marginal discrimination parameters for the general factor of the bifactor model [48]. Even though the unidimensional model showed some misspecification through residual correlation, the bifactor model demonstrated that most scale items loaded onto one factor. Therefore, we considered the misspecification ignorable, and the one-factor model was more parsimonious and had the most appropriate scale structure for the scale theory. Indeed, the correlation between the general factor of the bifactor model and the unidimensional model was 0.84. See Figs. 2 and 3.

**Table 6:** Confirmatory factor analysis: goodness of fit summary for the adapted GRMS frequency items

Goodness of fit indices	CFI	SRMR	RMSEA	loglikelihood	AIC	BIC
Model 1: Unidimensional graded response	0.9770	0.0750	0.1089	-1661.28	3063.7	3273.9
Model 2: Bidimensional graded response	0.9913	0.0492	0.0799	-1611.85	2994.0	3235.5
Model 3: Bifactor graded response	0.9882	0.0851	0.0504	-1549.64	3223.3	3467.7

*N* = 399

*CFI* Comparative fit index, *SRMR* Standardized root mean square residual, *RMESA* Root mean square error

**Table 7:** Unidimensional and bifactor graded response IRT model: discrimination and item location parameters

Item	Discrimination parameters: Unidimensional model					
	α	*b*_1_	*b*_2_	*b*_3_	*b*_4_	*b*_5_
I have felt unheard	3.02	-2.51	-3.56	-4.70	-5.83	-6.58
My comments have been ignored	3.83	-3.47	-4.84	-6.16	-7.16	-8.19
Some challenged my authority/autonomy	3.77	-4.83	-5.87	-7.25	-7.95	-9.29
I have been disrespected	4.62	-5.64	-7.09	-8.18	-9.17	-10.09
Someone has tried to “put me in my place”	6.74	-8.92	-10.61	-11.90	-13.63	-14.27
Felt excluded from services or resources	4.17	-5.69	-6.95	-8.11	-9.26	-9.94
Assumed I did not have much to contribute to conversation	4.57	-5.69	-7.10	-8.10	-8.92	-9.68
Someone told me told calm down	3.17	-3.10	-4.25	-5.32	-6.73	-8.20
Someone accused me of being angry when speaking calmly	3.70	-4.92	-5.67	-6.71	-8.09	-8.83
	Discrimination parameters: Bifactor model					
	α	*b*_1_	*b*_2_	*b*_3_	*b*_4_	*b*_5_
I have felt unheard	3.74	-3.09	-4.46	-5.95	-7.72	-8.62
My comments have been ignored	6.19	-26.28	-37.12	-47.28	-60.17	-67.19
Some challenged my authority/autonomy	3.63	-4.78	-5.72	-7.11	-8.37	-9.68
I have been disrespected	3.04	-5.69	-7.13	-8.40	-9.39	-10.44
Someone has tried to “put me in my place”	3.69	-20.62	-24.67	-27.81	-33.58	NA
Felt excluded from services or resources	3.40	-5.35	-6.40	-7.50	-8.31	-9.23
Assumed I did not have much to contribute to conversation	3.29	-4.87	-6.18	-7.33	-8.05	-8.73
Someone told me told calm down	2.23	-5.22	-7.06	-9.01	-11.0	-12.87
Someone accused me of being angry when speaking calmly	2.71	-4.63	-5.37	-6.26	7.77	-8.32

*N* = 399; α: item discrimination parameter; *b:* item location parameter. Marginal discrimination parameters presented

### Differential item functioning

We assessed the potential for differential item functioning by participant race and ethnicity, as well as language preference. We found similar response profiles among Black and Hispanic respondents, with no evidence of differential item functioning, see Table 8. Participants also responded similarly irrespective of language preference. We identified one item that differed by language, whether participants felt “excluded from service or resources”, which was more likely to be endorsed by Black than Hispanic respondents, likelihood ratio test, *p* = 0.025, adjusted for multiple comparisons.

**Table 8:** Evidence of differential item functioning by race and language using the likelihood ratio test

**Items**	**AIC**	**BIC**	**X2**	***df***	***P* value**
By Race (Black v. Hispanic)					
I have felt unheard		-	-	-	NA
My comments have been ignored	2.75	10.14	1.25	2	0.535
I have been disrespected	-0.94	6.45	4.94	2	0.254
Someone has tried to “put me in my place”	3.39	10.78	0.61	2	0.736
Felt excluded from services or resources	1.66	9.05	2.34	2	0.466
Assumed I did not have much to contribute to the conversation regarding my care	-	-	-	-	NA
Someone told me to calm down	-	-	-	-	NA
Someone accused me of being angry when speaking calmly	-	-	-	-	NA
Language (English v. Spanish)					
I have felt unheard	3.76	11.15	0.24	2	0.886
My comments have been ignored				-	NA
I have been disrespected	-0.73	6.67	4.73	2	0.188
Someone has tried to “put me in my place”	1.55	8.95	2.45	2	0.392
Felt excluded from services or resources	-6.15	1.25	10.15	2	0.025
Assumed I did not have much to contribute to the conversation regarding my care	-	-	-	-	NA
Someone told me to calm down	-	-	-	-	NA
Someone accused me of being angry when speaking calmly	-	-	-	-	NA

*P*-value adjusted for multiple comparisons using Bonferroni correction. Unestimated items were anchor items based on similarities in empirical distributions between comparison groups

### Criterion validity

Last, we computed Pearson correlations to assess the criterion validity of the GRMS. The GRMS total score was significantly and positively related to postpartum mental health outcomes as measured by the EPDS and GAD-7 scales, as shown in Table 9. We then conducted a subgroup correlation analysis to assess whether criterion validity differed across language race and ethnicity groups. GRMS was positively correlated with measures of postpartum mental health among Asian, Black, and Hispanic participants and among those who completed the survey in English and Spanish. Interestingly, as shown in Tables 2, 3, 4, 10 and 11, the association between postpartum mental health and GRMS was slightly weaker among Black participants and those who completed the survey in English compared to Spanish-speaking Hispanic participants, and among Asian participants there was a moderate association between GRMS and postpartum depression, but no association between GRMS and as anxiety.

**Table 9:** Correlations, means, and standard deviations among the GRMS, GAD-7, and EPDS measures

Measure	*n*	*M*	*SD*	1	2	3
1. GRMS	399	3.21	7.08	─		
2. GAD-7	399	3.96	4.35	.34***	─	
3. EPDS	399	5.01	4.16	.25***	.44***	─
4. Unidimensional model factor scores	399	0.55	0.49	0.88***	0.32***	0.30***
5. Bifactor general factor score	399	0.74	0.42	0.84***	0.31	0.30
6. Bifactor specific factor 1 factor score	399	0.27	0.49	0.45***	0.19	0.23
7. Bifactor specific factor 2 factor score	399	0.31	0.44	0.42**	0.19	0.23

**p < 0.005; ***p < .001

**Table 10:** Subgroup analysis, English language participants - correlations, means, and standard deviations among the GRMS, GAD-7, and EPDS measures

Measure	*n*	*M*	*SD*	1	2	3
1. GRMS	293	3.23	7.22	─		
2. GAD-7	293	4.04	4.52	.34***	─	
3. EPDS	293	4.81	4.17	.26***	.40***	─

***p < .001

**Table 11:** Subgroup analysis, Spanish language participants - correlations, means, and standard deviations among the GRMS, GAD-7, and EPDS measures

Measure	*n*	*M*	*SD*	1	2	3
1. GRMS	106	3.17	6.76	─		
2. GAD-7	106	3.91	3.83	.32***	─	
3. EPDS	106	5.29	3.82	.20***	.59***	─

***p < .001

## Discussion

With the growing interest in reporting instances of gendered racism experienced by Global Majority birthing people in hospital-based obstetric settings, the need for a simply administered tool is urgent. Given this, results from our analyses indicate that our adapted GRMS, created in collaboration with community-based birth justice advocates, is a valid measure for capturing Global Majority birthing people’s experiences of gendered racial microaggressions in an obstetric setting. Results from differential item functioning also support the validity of the scale for Black, Hispanic, and Spanish-speaking populations. Further, the results from the test information curve show that measurement reliability (i.e., information) was good among those with lower relative levels of gendered racial microaggression. As such, this tool performs well in hospital-based and clinical settings when Global Majority pregnant and birthing people’s medical encounters occur over a relatively short and finite period as opposed to lifetime experiences of microaggressions when we would expect the frequency of microaggressions to be higher. The scale had substantial floor effects whereby the modal response was no instances of gendered racial microaggression. This may reflect the population distribution of this construct, or that further work is needed to add items with high levels of informativeness at the upper end of experiences with gendered racial microaggression. Nonetheless, the IRT analysis supports the use of this scale among birthing people with lower levels of gendered racial microaggression.

We found evidence in our DIF analysis that Black and Hispanic participants similarly reported experiencing gendered racial microaggressions in obstetric settings. Research shows that people of the Global Majority often experience racism within healthcare systems [25, 49]. Birth equity scholars have identified racism as a cause of inequities in obstetric care and health outcomes among Global Majority birthing people [7, 50, 51]. Therefore, our finding that Black and Hispanic participants experienced gendered racial microaggressions in obstetric settings underscores the need for further research on understanding Global Majority birthing people’s experiences of racism in obstetric settings. We must conduct more research to highlight that discrimination and racism experienced by birthing people are not only limited to Black birthing people, but also differently experienced by other Global Majority populations to better understand the impact of gendered racial microaggressions in obstetric care regardless of race and ethnicity.

Similarly, we found evidence that the GRMS is suitable for use among English and Spanish-speaking participants, with few differences by language, and no identified differences by Black or Hispanic participants. As noted above, participants who took the survey in English were more likely to endorse being “excluded from service or resources,” than respondents who took the survey in Spanish. This finding aligns with previous research on obstetric and gendered racism that highlights the historical and ongoing contexts in which Black birthing people are often neglected, dismissed, and impeded from accessing knowledge and care [11, 30, 31].

Finally, our criterion validity subgroup analysis demonstrated stronger associations between experiencing gendered racial microaggressions and postpartum anxiety and depression among respondents who completed the survey in Spanish. This suggests that the translated GRMS appropriately captures experiences of subtle racism that are associated with negative mental health outcomes. Taken together, findings from our DIF analysis provide support for previous research and further evidence demonstrating the association between gendered racial microaggressions and negative postpartum mental health outcomes among English and speaking Spanish-speaking Black and Hispanic participants.

## Implications

The experience of racism and discrimination and its relationship to adverse obstetric outcomes, maternal health inequities, and postpartum mental health remains a crucial topic of investigation. Research indicates that birthing people across multiple racial and ethnic groups experience similarities and differences in the type of racism they face during pregnancy and birth impacting the health and wellbeing of themselves and their infants [25]. Our study provides evidence that gendered racial microaggressions in obstetric care extend beyond the well-documented experiences of racism among Black birthing people, and impact Global Majority birthing people with heterogeneous racial and ethnic identities who speak both Spanish and English. Results from our study illustrate the importance of using a measure that captures gendered racial microaggressions experienced by Global Majority birthing people in clinical settings.

In our correlational criterion validity analysis, we found correlations between GRMS and postpartum anxiety and depression. These findings align with both findings from Lewis & Neville’s finding that GRMS is related to psychological distress, and previous research highlights associations between Global Majority pregnant and birthing people’s experiences of racism during birth and postpartum depression [44, 45, 52]. Likewise, the subgroup analyses demonstrated that postpartum depression was associated with GRMS among Asian, Black, and Hispanic participants, and anxiety was associated with GRMS among Black and Hispanic participants, further supporting the validity of the GRMS in Global Majority populations. In addition, our findings underscore the need for clinical settings to address the occurrence of gendered racial microaggressions and their impact on postpartum mental health outcomes in care practices for Global Majority birthing people.

Our results strengthen the need for clinical settings to assess patient experiences of gender and racial discrimination among racially and ethnically diverse birthing populations. The short length of the nine-item GRMS allows for accessibility in capturing patient experiences of racism and discrimination during a limited hospital stay and as part of other efforts to understand patient satisfaction. Our study demonstrates the importance for healthcare professionals and hospital staff to be aware of Global Majority birthing peoples’ experiences of gendered racial microaggressions in obstetric settings and how these experiences may impact their postpartum mental health. It is important, however, for researchers and clinicians to not only capture and monitor experiences of gendered racial microaggressions but also to be accountable for these experiences by turning research into praxis. We suggest that further work be done to show the feasibility and implementation of adapting the scale to possibly be utilized as a performance indicator for those who provide care to Global Majority pregnant and birthing people in hospital and medical settings [53]. We also recommend that the GRMS, and other similar scales, be used to advance maternal equity. We suggest that results from such measures be used to implement new education and training on interpersonal interactions between patient and provider (e.g., empathy and antiracism training), as well as other departmental and institutional guidelines regarding care practices (e.g., quality improvement). In this way, we hope that such training will reduce the occurrence of gendered racial microaggressions, and Global Majority pregnant and birthing people will receive better quality of care and support from their providers.

## Limitations and future research

Despite the evidence suggesting our adapted GRMS scale measures the latent construct of gendered racial microaggressions among Global Majority birthing people, several limitations should be considered. First, we did not have information on participants' gender. Therefore, we were not able to assess how trans, non-binary, and individuals across the gender spectrum might differently experience gendered racial microaggressions. Participants, however, had the opportunity to disclose any additional information about themselves not captured in our survey. We recognize that pregnant and birthing people have different experiences with gendered racial microaggressions that are specific to their gender, for instance, they may experience transphobic and racialized microaggressions. Therefore, we recommend the development of a measure that captures gender diverse individuals’ experiences with gendered racial microaggressions during pregnancy and postpartum. Second, our sample did not include all racial and ethnic identities within the Global Majority population, and due to smaller sample sizes of participants who identified as Asian, Multiracial, and Other/Don’t Know, our DIF analysis was limited to participants who identified as Black and Hispanic. Although we could not include participants who identified as either Asian or Multiracial/Other in our formal validity analyses, the prevalence of GRM was (34.2%) among Asian participants and (44.2%) among Multiracial/Other participants. Our results are not generalizable to all birthing people within the Global Majority experiences with gendered racial microaggressions.

Lastly, there are a few limitations related to data collection and the scale itself. First, the survey was administered at one-time point at the participant’s bedside before being discharged after giving birth. Participants may have minimized the frequency in which they experienced gendered racial microaggressions given that they were still under the treatment and care of hospital staff when the survey was completed. Future research should explore administering GRMS at different periods and settings throughout pregnancy, birth, and postpartum to understand temporal and contextual influences. The data used for our analyses are cross-sectional, and we did not assess test–retest reliability. More research is needed to further assess the stability of the scale’s properties over time. Another drawback of this study was the observed floor effects in the GRMS not reported in the construction and validation of the original GRMS [18]. Further research is needed to understand the degree to which this property holds in other settings and uses. Nevertheless, these limitations do not diminish the importance of capturing experiences of gendered racial microaggressions in obstetric settings, nor the usefulness of a short nine-item scale too that can be used in a hospital setting among a racially and ethnically diverse Global Majority population that uses Spanish or English as a primary language.

## Conclusion

Our study expands on the Gendered Racial Microaggressions Scale by constructing and validating the adapted GRMS that captures a multiracial, multiethnic, and bilingual sample's experiences of gendered racial microaggressions in obstetric settings [18]. Results revealed that Global Majority birthing people who gave birth in a hospital setting experienced gendered racial microaggressions and that GRMS scores are correlated with existing anxiety and depression measures that are widely used in postpartum populations. The current study provides psychometric support for the newly developed GRMS measure, which was positively related to psychological distress during the postpartum period (e.g., postpartum depression and anxiety as measured by the GAD-7 and EPDS). The validation of our GRMS measure provides a significant contribution to current efforts to capture and combat various experiences of racism among birthing people of the Global Majority in clinical settings. We recommend that clinicians and practitioners, medical professionals, and other healthcare workers can use this validated tool to assess and intervene on the effects birthing people of the Global Majority face, such as postpartum mental health consequences, when experiencing gendered racial microaggressions in an obstetric setting.

## Data Availability

The datasets used and/or analyzed during the current study are available from the corresponding author on reasonable request.
